# 33 Enhancing Healthcare Professionals’ Competencies in Physical Activity: The Implementation of the vAdBeCeDa®- a multicomponent exercise programme to prevent falls and frailty in elderly

**DOI:** 10.1093/eurpub/ckae114.012

**Published:** 2024-09-26

**Authors:** Maja Dolenc, Tjaša Knific, Suzana Pustivšek, Katja Tomažin

**Affiliations:** University of Ljubljana, Faculty of Sport, Ljubljana, Slovenia; National institute for public health Slovenia, Ljubljana, Slovenia; National institute for public health Slovenia, Ljubljana, Slovenia; University of Ljubljana, Faculty of Sport, Ljubljana, Slovenia

## Abstract

**Purpose:**

Longitudinal studies of multicomponent exercise programs have produced encouraging results suggesting that sustained physical activity (PA) has a significant impact on the fall prevention and frailty in elderly. Promoting lifelong PA for elderly requires a community-based approach and effective collaboration between the health and sports professionals. The National Institute of Public Health in Slovenia conducts activities as part of the four-year project “Integration of Geriatric Care for the Elderly” In 2024, a series of educational events entitled “Supporting the Elderly - Challenges in Geriatric Care for the Elderly in Slovenia” took place in various locations in Slovenia. The aim of these events was to improve the competences of health, sports and social care professionals involved in the care of the elderly and to promote their collaboration.

**Project description:**

In the series of events we have addressed more than 1000 participants, with the results showing promising trends in the evaluation of participants’ satisfaction and the usefulness of the topics for their regular work. Our aim was to train professionals through the use of modern information and communication technology (ICT). The training was delivered in a hybrid format that included recorded lectures, online presentations and practical live workshops. We addressed the prescription of PA and exercise as one of the most promising motivating factors for habit change in elderly. The vAdBeCeDa®, i.e., a novel multi-component exercise program, developed in collaboration between sports, health and fitness professionals, was presented as part of the PA promotion. The program aims to prevent falls and frailty in community-dwelling elderly by improving strength, stability, balance, flexibility and walking speed through targeted exercises. It is in line with the WHO guidelines on PA and physical inactivity and the latest global recommendations for fall prevention in elderly.

**Conclusions:**

The vAdBeCeDa® program will be implemented in the existing network of Health Promotion Centers (HPC) and Healthy Clubs (HC) throughout the country so that the entire elderly population can exercise at the expense of the Slovenian health insurance. Recognizing that strengthening the competencies of healthcare professionals is an essential prerequisite for ensuring quality care for elderly.

